# The Quality of Sputum Smear Microscopy in Public-Private Mix Directly Observed Treatment Laboratories in West Amhara Region, Ethiopia

**DOI:** 10.1371/journal.pone.0123749

**Published:** 2015-04-07

**Authors:** Almaw Manalebh, Meaza Demissie, Daniel Mekonnen, Bayeh Abera

**Affiliations:** 1 USAID-Ethiopia, Private Health Sector Programme, Bahir Dar, Ethiopia; 2 Addis Continental Institute of Public Health, Gondar, Ethiopia; 3 Departments of Medical Microbiology, Parasitology and Immunology, College of Medicine and Heath Sciences, Bahir Dar University, Bahir Dar, Ethiopia; Public Health Research Institute at RBHS, UNITED STATES

## Abstract

Ethiopia adopted Public-Private Mix Directly Observed Treatment Short Course Chemotherapy (PPM-DOTS) strategy for tuberculosis (TB) control program. Quality of sputum smear microscopy has paramount importance for tuberculosis control program in resource-poor countries like Ethiopia. A cross-sectional study was conducted to assess the quality of sputum smear microscopy in 37 Public-Private Mix laboratories in West Amhara, Ethiopia. The three external quality assessment methods (onsite evaluation, panel testing and blind rechecking) were employed. Onsite assessment revealed that 67.6% of PPM-DOTS laboratories were below the standard physical space (5 X 6) m^2^. The average monthly workload per laboratory technician was 19.5 (SD±2.9) slides with 12.8% positivity rate. The quality of Acid Fast Bacilli (AFB) staining reagents was sub-standard. The overall agreement for blind rechecking of 1,123 AFB slides was 99.4% (Kappa = 0.97). Reading of 370 AFB panel slides showed 3.5% false reading (Kappa = 0.92). Moreover, the consistency of reading scanty bacilli slides was lower (93%) compared to 1+, 2+ and 3+ bacilli. Based on blind rechecking and panel testing results, PPM-DOTS site laboratories showed good agreement with the reference laboratory. Physical space and qualities of AFB reagents would be areas of intervention to sustain the quality of sputum smear microscopy. Therefore, regular external quality assessment and provision of basic laboratory supplies for TB diagnosis would be the way forward to improve the quality of sputum smear microscopy services in PPM-DOTS laboratories.

## Introduction

Tuberculosis (TB) is a chronic infectious disease caused by *Mycobacterium tuberculosis* [1]. The main source of infection is untreated smear-positive pulmonary tuberculosis patients discharging *M*. *tuberculosis*. In Ethiopia, tuberculosis is a major public health problem; therefore, health facility laboratories are integrated in the national TB control program [1, 2]. Ethiopia adopted the ‘Stop TB’ strategy’ of World Health Organization (WHO), which is Public-Private Mix Directly Observed Treatment (PPM-DOTS) in 2006 [3], but DOTS was limited only to government sector health facilities. In Ethiopia, private health sectors have employed 55% of general practitioners, 65% of specialists and 79% of laboratory technicians [4]. Expanding TB care services to the private sector has increased access for clients who are reluctant to visit public health facilities due to fear of stigma and perception of unsatisfactory quality of health care services [5].

Sputum smear microscopy is a key component of DOTS in developing countries like Ethiopia [6]. Thus, in countries with a high prevalence of TB, sputum smear microscopy remains the most cost-effective tool for diagnosing and treatment of TB [3]. However, it detects only 20.5% to 74.2% of culture positive TB cases [7, 8], and functions well in quality assured system to produce accurate, reliable and reproducible laboratory results [3].

In Ethiopia, according to the assessment conducted in 2011, 317 PPM-DOTS facilities provide TB diagnosis and treatment services [9]. The highest number of PPM-DOTS facilities (28.4%) was found in Amhara National Regional State, where this study was conducted. In Ethiopia, between August 2006 to and July 2011, 61,525 (9.1%) TB cases were diagnosed in private health facilities [4].

Poor quality sputum microscopy services may result in failure to detect persons with active tuberculosis and unnecessary anti-TB treatment for non-TB cases. In addition, errors in reading sputum microscopy may result in prolonged treatment, or unnecessary treatment termination which predisposes the development of drug resistant tuberculosis (MDR-TB) [6, 10].

Various factors including infrastructure, work experience in sputum smear microscopy and reagents supplies affect the quality of sputum smear microscopy [11]. There is no documented information on the quality of sputum smear microscopy in PPM-DOTS site laboratories in the country especially in the study area. Therefore, this study was conducted to assess the quality of sputum smear microscopy in public-private mix laboratories in West Amhara, Ethiopia.

## Materials and Methods

A cross-sectional study was carried out in six administrative zones in July 2013, West Amhara, Ethiopia. This study included 4 general hospitals, 11 higher clinics and 22 medium clinics. Medium and higher clinics were categorized under primary health care unit in private health sectors. Their service package was mainly basic curative services. Medium clinics were staffed by medium level health professionals, while higher clinics were staffed by higher level medical specialists. From 42 PPM-DOTS sites, 37 were included in this study, but the other 5 sites (4 medium and 1 higher clinics) were excluded using exclusion criteria.

### Study sites

The study sites included 37 Private—Public DOTS site laboratories that provide TB diagnostic service in Western Amhara.

### Inclusion and Exclusion Criteria

All PPM-DOTS site laboratories providing sputum smear microscopy service were included. However, PPM-DOTS sitelaboratories providing sputum smear microscopy service less than one year were excluded.

### Slide sample size determination and sampling

AFB slide sample size for blind rechecking was calculated using the Lot Quality Assurance Sampling (LQAS) method and the national TB external quality assurance (TBEQA) guidelines [10]. Thus, 1123 AFB slides were determined to run blind rechecking. The numbers of slides from each facility were taken based on annual negative slide volume (ANSV) and slide positivity rate (SPR) [10]. Slides from each PPM-DOTS site laboratory were selected by systematic random sampling method from a list of sputum samples processed between January 1, 2013 and June 30, 2013 in TB laboratory log books.

### Data Collection tools and procedures

External quality assessment methods (blinded rechecking, panel testing and onsite evaluation) were used to assess the quality of sputum smear microscopy in PPM-DOTS sites.

### Blind rechecking of AFB slides

Slides for random blind rechecking were selected according to PPM-DOTS site, AFB positivity slide rate and annual AFB negative slides [10]. Randomly selected slides were kept in slide boxes and then taken to Bahir Dar Regional Health Research Laboratory Center (BRHRLC) for blind rechecking. Each slide was evaluated according to the national External Quality Assurance (EQA) guideline [10]. The slides were evaluated and re-read by the first controller and results were compared with results of PPM-DOTS site laboratories by quality officer. Divergent results were verfied by second controller without sharing the first controller’s results. The second controller reading results were considered as final [10,12].

### AFB panel sample preparation

AFB-positive and negative sputum samples were collected from samples sent to BRHRLC for TB sputum culture. Seven known good quality negative sputum samples (>20 white blood cells /field) was pooled and then split into 3 ml aliquots using eight 50ml falcon tubes. The sputum was checked again for AFB after pooling using Ziehl-Neelsen and Florescent microscopy. Three positive sputum samples with grade 2+ and 3+ were pooled for positive AFB panels.

From each tube containing 3ml sputum, 150μl of 37%w/v formaldehyde was mixed and incubated for one hour at ambient temperature (25°C). After 1 hour, 1ml of 4% NaOH (petroff method) was added and vortexed for 5 minutes. Then, 20ml of sterile distilled water was added. The whole batch was then incubated at 60°C in water bath and mixed by inverting the tubes for 10 minutes for negative sputum and 30 minutes for positive sputum. After the incubation, distilled water was added to make a total volume of 40 ml. Finally, it was centrifuged at 3,000 x g for 20 minutes 4°C. Pellets from each negative test tube were recombined into one test tube and the positive pellets into another test tube. Then, the mixtures were re-suspended in 2 ml sterile distilled water [12].

### Preparation of bacilli density

The stock solution of positive AFB sputum pellet was diluted with the negative stock sputum pellet. The dilution factor was calculated using the following formula: N = (DC/AC) × A, where N is the number of drops of positive sputum to be added, DC is the desired AFB concentration, AC is the actual AFB concentration and A is the number of drops in a given volume [13]. AC was obtained from a smear of two drops of the positive stock solution. The positive stock had 50–60 AFB per microscope fields. The actual numbers of bacilli were: ≥10 AFB/field for 3+ smears, 5 AFB/field for 2+ smear, 50 AFB/100 fields for 1+ smear, and 5 AFB/100 fields for scanty (1-9AFB/100 field) smear. Six slides were prepared from each grade and given to six laboratory technologists for validating consistencies. All readings (3+, 2+, 1+ and scanty) were the similar among the six readers with ±2 SD. For panel testing, AFB stained sputum smears (n = 185) and unstained smears (n = 185) were prepared [10]. Stained AFB slides (one 2+, one 1+, one negative and two scanty) and unstained (one 3+, one 1+, one scanty and two negative) panels with second degree of difficulty were prepared. These stained and unstained smears were separately packed and safely transported into each PPM-DOTS site.

### Statistical analysis

Data were analyzed using the Statistical Package for Social Sciences version 20 software (IBM Corp. Released 2011. IBM SPSS Statistics for Windows, Armonk, NY: IBM Corp). The agreement in reading between the PPM-DOTS site laboratories and the reference laboratory readings were analyzed using kappa statistics. The results were interpreted as poor agreement (K < 0.20), Fair agreement (K = 0.20 to 0.39), moderate agreement (K = 0.40 to 0.59), Good agreement (K = 0.60 to 0.79) and very good agreement (K = 0.80 to 1.00).

### Definitions of terms as used in the national guideline [10]

High False Negative (HFN) = A 1+ to 3+ positive smear that is misread as negative.

High False Positive (HFP) = A negative smear that is misread as 1+ to 3+ positive.
Low False Negative (LFN) = A scanty (1–9 AFB /100 fields) positive smear that is misread as negative.
Low False Positive (LFP) = A negative smear that is misread as a scanty (1–9 AFB / 100 fields) positive.
Major Error = The most critical error which has the highest potential impact on patient management, and can result in an incorrect diagnosis or improper management of a patient.
Minor Error = Errors that may have some impact on patient management. This type of error is considered less serious in evaluating laboratory performance.
Panel Testing = Sending stained and/or unstained smears from the reference laboratory to microscopic centers to check proficiency in reading and reporting.

“The second degree of difficulty in reading AFB panel slides” includes 1 slide with graded 3+and graded 2+, 2 slides graded 1+, 3 slides graded 1–9 bacilli /100 fields and 3 negative slides. These packages of slides were prepared to challenge the laboratory technicians’ reading skill.

### Ethical considerations

The study was ethically cleared by the Research and Ethics Review Board of the University of Gondar. The letter of support written by Research and Ethics review Board was submitted to Amhara Regional Health Bureau. Then, officials at all levels were communicated through formal letters from the Regional Health Bureau to private health facilities (study sites). Prior to data collection, written consent was obtained from the heads of each health facility and the head of laboratory personnel. Furthermore, written consent was also obtained from laboratory professionals who participated in interview and panel testing. Confidentiality was maintained by coding each facility starting from data collection to analysis.

## Results

### Onsite assessment

A total of 18,267 sputum smears were processed in 37 PPM- DOTS site laboratories in one year. The average number of smears performed per laboratory personnel per month was 19.5 (SD±2.9).The overall slide positivity rate was 2,340 (12.8%), and the least positivity rate (616 /9.4%) was observed in medium clinics Among 78 laboratory professionals, 3.8% were MSc holders, 12.8% senior laboratory technologists and 83.3% were laboratory technicians with diploma (grade 12+2). The majority of laboratory professionals (88.5%) had above two years of work experience.

Assessment of the laboratory infrastructure showed that 21 (56.8%) sites of PPM-DOTS provided service in rental house which were built for hotels. Regarding the laboratory room size, 67.6% of PPM-DOTS site laboratories had room size below the standards (5 x 6) m^2^. Of these, 4 (10.8%) PPM-DOTS laboratories had room sizes less than (3 x 4) m^2^. Moreover, 15 (40.5%) had no water supply in the laboratory. Assessment of waste disposal system showed that, 10.8% of the laboratories had a poor waste disposal system and 35.1% had no waste containers with lids.

Regarding reagents for acid fast staining, the private participant facilities claimed that the sources of laboratory reagents were not up to the standards as of public facilities. For instance, 22 (59.5%), 10 (27%) and 5 (13.5%) of PPM-DOTS sites get carbolfuchsin from non-licensed, licensed/government and unknown providers. None of the participant facilities collected reagents from the nearby Bahir Dar Regional Health Research laboratory Center. Moreover, in 35 (94.6%) PPM-DOTS’ reagents were not correctly labeled with the expiry date. Internal quality control was critical for those facilities which had no genuine provider of AFB reagents. Thus, 20 (54%) of the participant laboratories practiced internal quality control but only 16 (43.2%) of them used the national guideline regularly.

### Blinded rechecking of slides

A total of 1,123 slides (n = 161 positive and n = 962 negative) were collected for blind rechecking from all the participant PPM-DOTS site laboratories. Of these, 895 (79.7%) had good quality of staining, 835 (74.4%) had proper smear size and 793 (70.6%) had good smear thickness. Discordant results were observed from 5 (13.5%) of the PPM-DOTS site laboratories. The overall sputum smear discordant result was 7 (0.6%). The proportion of positive and negative slides read correctly is depicted in Table 1. The overall sensitivity and specificity were 98% and 99.6%, respectively. The levels of agreement between reference laboratory and PPM-DOTS site laboratories were good (Kappa = 0.97) (Table 1).

**Table 1:** Results of blind rechecking of Acid Fast Bacilli slides by levels of PPM-DOTS site Laboratories.

**Variables**	**Hospital**	**Higher Clinic**	**Medium Clinic**	**Total**
**Total number of slides**	112	255	756	1123
**Number of negative slides**	97	204	662	963
**Number of positive slides**	15	51	94	160
**Good quality of staining**	84 (75)	218 (85.5)	593 (78.4)	895 (79.7)
** Proper smear Size**	56 (50)	208 (81.6)	571 (75.5)	835 (74.4)
** Proper thickness**	75 (67)	192 (75.3)	526 (69.6)	793 (70.6)
**Sensitivity**	100	96	99	98
**Specificity**	100	98.5	99.8	99.6
**Positive predictive value**	100	93.2	99	97.5
**Negative predictive value**	100	99	99.8	99.7
**High false negative**	0	2(0.8)	0	2 (0.2)
**High false positive**	0	3 (1.2)	1 (0.1)	4 (0.4)
**Quantification error**	1 (0.9)	2 (0.8)	4 (0.5)	7 (0.6)
** Major error** *	0	5 (2.0)	1 (0.1%)	6 (0.5)
** Agreements**	112 (100)	250 (98)	754 (99.7)	1116 (99.4)
**Kappa Value**	1	0.94	0.99	0.97

* ^Major error includes both high false positive and high false negative errors. Numbers in brackets are percentages.^

### Panel testing

A total of 370 proficiency panel testing slides (n = 111 negative and n = 259 positive) with different bacilli densities were read by 37 laboratory personnel working in private laboratories. Of these, 12 laboratory technicians (32%) reported different types of errors. The consistency level of readers was 96% from all panel test slides. However, the level of consistency was different between scanty panel slides and other grade slides (Table 2).

**Table 2:** Consistency of results in different grades of Acid Fast Bacilli panel test slides.

**PPM-DOTS site results**	**Panel test results**					Total
	Negative	Scanty	1+	2+	3+	
**Negative**	107	7	2	0	0	116
**Scanty**	1	47	16	0	1	65
**1+**	3	54	40	7	4	108
**2+**	0	3	16	11	11	41
**3+**	0	0	0	19	21	40
**Total**	111	111	74	37	37	370
**Agreement in percent**	96	93	97	100	100	96

In this study, 62% errors were observed from unstained panel smears, whereas 38% errors were from stained panel smears (Table 3). Comparison of panel test reading results by level of health facilities, and overall sensitivity, specificity, positive predictive value (PPV) and negative predictive value (NPV) is presented in Table 4. The agreement of panel results was very good (Kappa = 0.92).

**Table 3:** Reading results of stained and unstained sputum AFB panel testing.

**Types of panel slides**	**Major error**		**Minor error**			**Total error**
	HFN	HFP	LFN	LFP	QE	
**Stained panel slide**	1	1	3	0	3	8 (38%)
**Unstained panel slide**	0	2	5	1	5	13 (62%)
**Total**	1	3	8	1	8	21 (100%)

^HFN, High False Negative; HFP, High False Positive; LFN, Low False Negative; LFP, Low False Positive; QE, Quantification Error^

**Table 4:** Panel testing performance by levels of PPM-DOTS site Laboratory.

**Parameter**	**Hospital**	**Higher Clinic**	**Medium Clinic**	**Total**
**Total panel slides**	40	110	220	370
**Total negative panel**	12	33	66	111
**Total positive panel**	28	77	154	259
**High false negative**	0	0	1 (0.5)	1 (0.3)
**High false positive**	0	0	3 (1.4)	3 (0.8)
**Low false negative**	0	4 (3.6)	4 (1.8)	8 (2.2)
**Low false positive**	0	0	1 (0.5)	1 (0.3)
**Quantification Error**	1(2.5)	4(3.6)	3 (1.4)	8 (2.2)
** Major Error**	0	0	4 (1.8)	4 (1.1)
**Sensitivity**	100	94.8	96.7	96.5
**Specificity**	100	100	93.9	96.4
**Positive predictive value**	100	100	97.4	98.4
**Negative predictive value**	100	89.1	92.5	92.2
**Percentage agreement**	100	96.4	95.9	96.5
**Kappa Value**	1	0.916	0.903	0.92

^Major error: includes both high false positive and high false negative errors.^

## Discussion

In Ethiopia, data on quality of sputum smear microscopy particularly in public-private laboratories is limited. This study, therefore, provides important information for the improvement of quality of sputum microscopy services. The findings indicated that only 32.4% of laboratories meet the laboratory room size standards of the national TB guideline [4]. Moreover, 10.8% of the laboratory rooms were below 3 x 4 m^2^, which have limited space for ventilation and laboratory processing for TB diagnosis. This could be because 56.8% PPM-DOTS sites provided their service in rental houses and this could hinder the sites from meeting the standard required of a laboratory. The sources of reagents were not up to the standards of laboratory supplies for sputum microscopy because none of the laboratories collected reagents from Regional Laboratory. This result is different from the results obtained from other African countries. For instance, in Ghana, 94% AFB reagents were obtained from the Regional Laboratory [14].

The average workload of sputum microscopy was 19.5 smears per month per laboratory professional. This is in line with a study conducted in Uganda [15]. However, the average number of smear in this study was by far lower than the recommended range of 20 to 25 smears per laboratory [12]. It is also lower than the reports from high TB-incidence countries such as Malawi which documented an average number of smears performed per working day of 6.0 to 4.0 [16]. The proportion of smear positivity rate was 12.8% which was between the recommendations of the national guideline (5% to 15%). This reflects good screening process of TB suspected patients and indicates acceptable efficiency of smear microscopy. The smear positivity rate in this study was lower than the rate in Tanzania (19%), Malawi (17%), Senegal (19%) and Benin (32%) [16].

### Blind rechecking of slides

In the private-public AFB microscopy, the proportion of good staining quality, proper thickness and proper smear size was concordant with studies conducted in Ghana, Tanzania and Taiwan [17–19]. However, thickness of the smear was below the standard of the smear quality evaluation criteria. This might be because of failure to use purulent sputum from patients; that is, patients might have submitted saliva in place of sputum. Moreover, we observed that the majority of laboratories used local made smearing material like Dengel (Amharic equivalent for papyrus) or Zembaba (palm) stick (instead of applicator sticks and/or wire loop) that might not be appropriate for picking the purulent part of sputum.

The overall degree of false reading in the present study was 3.2% (Kappa = 0.936). This is lower than a study conducted in Tanzania, which reported 10.8% false reading (15). Likewise, studies from India, Malawi and Columbia reported 1.3 to 3.7% false readings [20–22]. The overall sensitivity and specificity in the present study were better than the studies conducted in southern Ethiopia [23] and Tanzania [19]. Similarly, a study in Mexico showed 98.0% sensitivity and 99.7% specificity [13]. Thus, based on blind reaching, the levels of agreement in private- laboratories with the reference laboratory were within the acceptable ranges.

### Panel testing

Known panel of AFB slides of different smear status was used to assess the reading proficiency of laboratory personnel. Reading errors were committed on negative, scanty (1–9 AFB/100 fields) and positive (1+) slides. The overall level of consistency in scanty, negative and positive (1+) panel slides was 96%. This is similar with a studies conducted in India and Mexico [13, 24, 25]. However, it is higher than a study conducted in Bangalore which reported 80% consistency level [26].

Errors were reported in all levels of health facilities except in general hospital laboratories. All major errors were observed in medium clinics. From major errors, three high false positive (HFP) errors were reported in one clinic and one high false negative (HFN) was observed in another clinic. The possible root cause was competency of technicians which needs to be improved. HFP and HFN are the major errors which are considered as gross errors that result in unnecessary treatment for non-Tb cases and misclassification of TB cases for proper treatment of TB patients. Any major error most likely causes unacceptable performance and requires corrective action. This study has revealed HFP and HFN which are comparatively lower than the previous study conducted in India [25].

The majority of errors (62%) were reported from unstained panel smears. All quantification errors were under-grade from unstained panel smears. This difference could be explained by poor quality of AFB reagents used in PPM-DOTS sites. Because, onsite assessment result showed that the sources and quality of AFB reagents were not up to the standards for public-private laboratories.

The present study has the following strengths. The sample size for PPM-DOTS site laboratories was optimal, and three quality assessment methods were used to triangulate the results. The main limitations of this study were selection bias which is unavoidable for random blind rechecking slides, and non-inclusion of public health facilities for comparison purpose.

## Conclusions

The performance of PPM-DOTS site laboratories on blind rechecking and panel test reading showed good agreement with the reference laboratory. The overall false reading on blind rechecking and panel testing results were not different from the levels of PPM-DOTS site laboratories. The quality and sources of AFB reagents were below the standards in private DOTS laboratories; thus, they accounted for poor quality of sputum smear microscopy. This is supported by panel testing assessment because most errors were observed from unstained panel smears. The implementation of external quality assessment and internal quality control was not regularly practiced.
